# Ca^2+^, Astrocyte Activation and Calcineurin/NFAT Signaling in Age-Related Neurodegenerative Diseases

**DOI:** 10.3389/fnagi.2018.00199

**Published:** 2018-07-09

**Authors:** Pradoldej Sompol, Christopher M. Norris

**Affiliations:** ^1^Sanders-Brown Center on Aging, University of Kentucky College of Medicine, Lexington, KY, United States; ^2^Department of Pharmacology and Nutritional Sciences, University of Kentucky College of Medicine, Lexington, KY, United States

**Keywords:** Alzheimer’s disease, Ca^2+^, glia, dementia, astrocytes, neuroinflammation, synapse

## Abstract

Mounting evidence supports a fundamental role for Ca^2+^ dysregulation in astrocyte activation. Though the activated astrocyte phenotype is complex, cell-type targeting approaches have revealed a number of detrimental roles of activated astrocytes involving neuroinflammation, release of synaptotoxic factors and loss of glutamate regulation. Work from our lab and others has suggested that the Ca^2+^/calmodulin dependent protein phosphatase, calcineurin (CN), provides a critical link between Ca^2+^ dysregulation and the activated astrocyte phenotype. A proteolyzed, hyperactivated form of CN appears at high levels in activated astrocytes in both human tissue and rodent tissue around regions of amyloid and vascular pathology. Similar upregulation of the CN-dependent transcription factor nuclear factor of activated T cells (NFAT4) also appears in activated astrocytes in mouse models of Alzheimer’s disease (ADs) and traumatic brain injury (TBI). Major consequences of hyperactivated CN/NFAT4 signaling in astrocytes are neuroinflammation, synapse dysfunction and glutamate dysregulation/excitotoxicity, which will be covered in this review article.

## Introduction

The central role of Ca^2+^ dysregulation in age-related memory deficits and neurodegenerative disease, proposed more than 30 years ago (Gibson and Peterson, 1987; Khachaturian, 1987; Landfield, 1987; Abdul et al., 2009), has been supported time and again by molecular, electrophysiological, biochemical and behavioral studies and is the subject of many excellent reviews (Alzheimer’s Association Calcium Hypothesis Workgroup, 2017; Frazier et al., 2017; Gibson and Thakkar, 2017; Pchitskaya et al., 2018). Neurons are often the focus of studies on Ca^2+^ dysregulation, and for good reason. Ca^2+^ signaling is an absolutely essential mechanism for both intra- and interneuronal communication. Moreover, disruption of any of the many neuronal Ca^2+^ regulatory checkpoints can lead to the structural deterioration of neurons and neuronal death, which are defining features of most neurodegenerative diseases. Nonetheless, it is becoming increasingly clear that Ca^2+^ dysregulation underlies altered function and viability of other non-neuronal cells during aging and disease, especially astrocytes. Several recent articles have provided comprehensive reviews of Ca^2+^ signaling mechanisms and Ca^2+^ dyregulation in astrocytes as a function of disease (Vardjan et al., 2017; Verkhratsky et al., 2017; Zorec et al., 2018). The following review will instead focus on the protein phosphatase calcineurin (CN) as an emerging mechanism for linking astrocytic Ca^2+^ dysregulation to neuroinflammation, glutamate dysregulation, amyloid pathology and synaptotoxicity. Particular emphasis will be placed on CN interactions with the nuclear factor of activated T cells (NFATs), though other CN-sensitive transcription factors such as nuclear factor κB (NFκB) and forkhead O3 (FOXO3) will also be considered.

## Ca^2+^ Dysregulation in Activated Astrocytes

Astrocytes are abundant and versatile cells that play critical roles in brain metabolism, vascular regulation, interneuronal signaling and defense. Fundamental to many of these duties are Ca^2+^ ions, which are handled by a sophisticated network of plasma membrane channels, Ca^2+^ pumps, Ca^2+^ binding proteins and intracellular stores (for recent comprehensive reviews see; Rusakov, 2015; Bazargani and Attwell, 2016; Shigetomi et al., 2016; Guerra-Gomes et al., 2017). Together, these mechanisms, and others, coordinate dynamic Ca^2+^ responses (e.g., Ca^2+^ waves and sparks) that can be propagated within the confines of individual astrocytes and also across large astrocyte syncytia via interconnecting gap junction channels (De Bock et al., 2014; Zheng et al., 2015; Fujii et al., 2017). The recent application of three-dimensional multiphoton imaging to astrocyte Ca^2+^ transients has highlighted the complexity and heterogeneity of Ca^2+^ signaling within different astrocyte compartments (e.g., soma, processes and endfeet) and perhaps points to an approaching renaissance in our understanding of the role of astrocytes in brain function and disease. Astrocytic Ca^2+^ dysregulation appears to be indelibly linked to morphologic transformations (i.e., astrocyte “activation” or “reactivity”) characterized by hypertrophic somata and processes and upregulation of the intermediate filament protein, GFAP (Pekny and Nilsson, 2005; Sofroniew, 2009; Rodríguez-Arellano et al., 2016; Bindocci et al., 2017). Astrocyte activation is triggered by a diverse range of injurious stimuli and is frequently localized to regions of frank pathology (e.g., damaged blood vessels, necrotic tissues and protein aggregates). Along with activated microglia, activated astrocytes provide one of the best neuroanatomical hallmarks of neuroinflammation.

Immunohistochemical studies have revealed the upregulation of numerous Ca^2+^ signaling mediators in activated astrocytes including: Ca^2+^ related proteases (Shields et al., 1998, 2000; Feng et al., 2011), L-type voltage-sensitive Ca^2+^ channels (Xu et al., 2007, 2010; Willis et al., 2010; Daschil et al., 2013; Wang et al., 2015), transient receptor potential vanilloid channels (Shirakawa et al., 2010; Butenko et al., 2012), endoplasmic reticulum Ca^2+^-release channels and Ca^2+^ pumps (Grolla et al., 2013), Ca^2+^-dependent K^+^ channels (Yi et al., 2016), and Ca^2+^ binding proteins (McAdory et al., 1998). Most extracellular factors that promote robust astrocyte activation *in vivo* (e.g., cytokines, reactive oxygen species, protein aggregates, excitotoxins, …etc) also trigger Ca^2+^ dysregulation (e.g., elevated Ca^2+^ levels, augmented Ca^2+^ transients) in primary culture and brain slices (Sama and Norris, 2013). Similar functional indices of Ca^2+^ dysregulation have been noted in animal models of AD (Takano et al., 2007; Kuchibhotla et al., 2009; Delekate et al., 2014), brain edema (Thrane et al., 2011), stroke (Ding et al., 2009; Rakers and Petzold, 2017) and epilepsy (Ding et al., 2007; Tian et al., 2010). The relationship between Ca^2+^ dysregulation and astrocyte activation is very likely to be bi-directional in nature. Indeed, Ca^2+^ modulates the activity of numerous transcription factor pathways (Mellstrom et al., 2008), several of which (e.g., NFκB, JAK/STAT, FOX proteins, peroxisome proliferator-activated receptors (PPARs) and activator protein-1 (AP-1), among others) have been implicated in shaping gene expression programs involved in astrocyte activation (Perez-Nievas and Serrano-Pozo, 2018). So, once astrocytic Ca^2+^ dysregulation is set in motion by injurious and/or neuroinflammatory factors, there are multiple routes through which Ca^2+^ could maintain astrocytes in an activated state. Perhaps the most direct link between Ca^2+^ and the gene regulatory machinery in astrocytes (and most other cell types) is provided by NFAT transcription factors, which are directly activated by the Ca^2+^-dependent protein phosphatase, CN. Mounting evidence, discussed below, shows that CN/NFATs exhibit clear signs of hyperactivation, and/or increased expression, in subsets of activated astrocytes, while cell-specific targeting approaches suggest that CN/NFAT signaling drives or exacerbates multiple forms of neuropathology.

## CN Dysregulation and Neurodegenerative Disease

CN is a highly abundant protein found throughout the brain, appearing at high levels in neurons and low levels in glia in healthy adult animals (Goto et al., 1986a,b; Polli et al., 1991; Kuno et al., 1992). Hyperactive CN signaling is observed in human postmortem brain tissue at early stages of cognitive decline associated with AD, ramping up in later disease stages in parallel with worsening amyloid pathology, neurofibrillary pathology and/or cognitive decline (Liu et al., 2005; Abdul et al., 2009; Wu et al., 2010; Mohmmad Abdul et al., 2011; Qian et al., 2011; Watanabe et al., 2015; Pleiss et al., 2016b). Other human neurodegenerative conditions associated with increased CN signaling include Parkinson’s disease (Caraveo et al., 2014), dementia with Lewy bodies (Martin et al., 2012; Caraveo et al., 2014) and vascular pathology (Pleiss et al., 2016b). Similar changes are often recapitulated to a significant degree in corresponding animal models of aging and neurodegeneration (Foster et al., 2001; Huang et al., 2005; Norris et al., 2005; Shioda et al., 2006; Reese et al., 2008; Mukherjee et al., 2010; Wu et al., 2010; D’Amelio et al., 2011; Martin et al., 2012; Rosenkranz et al., 2012; Furman et al., 2016; Sompol et al., 2017). Moreover, inhibition of CN signaling with the commercial immunosuppressant drugs, tacrolimus and cyclosporine, commonly imparts neuroprotection in experimental models of injury and disease (Kuchibhotla et al., 2008; Wu et al., 2010; Rozkalne et al., 2011; O’Donnell et al., 2016; Xiong et al., 2018), reduces neuroinflammation (Yoshiyama et al., 2007; Rojanathammanee et al., 2015; Fields et al., 2016; Manocha et al., 2017a; Shah et al., 2017), improves synapse function (Chen et al., 2002; Dineley et al., 2010; Cavallucci et al., 2013; Kim et al., 2015), inhibits cognitive loss (Taglialatela et al., 2009; Dineley et al., 2010; Kumar and Singh, 2017; Liu et al., 2017), and may even extend lifespan (Yoshiyama et al., 2007). Consistent with the animal literature, an epidemiological investigation found that daily tacrolimus use reduced the risk of dementia in kidney transplant patients relative to age-matched healthy individuals in the general population (Taglialatela et al., 2015).

**Figure 1:**
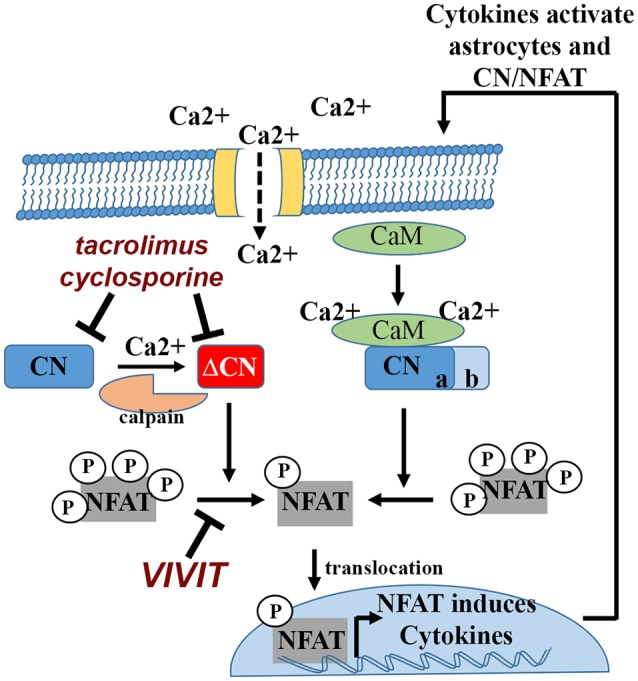
Calcineurin (CN)/nuclear factor of activated T cells (NFATs) signaling in astrocytes and bidirectional interactions with cytokines. Cytokines and other inflammatory factors lead to Ca^2+^ elevations in astrocytes. Ca^2+^ binds to calmodulin (CaM), which in turn, binds to and activates CN. CN dephosphorylates NFAT transcription factors, leading to nuclear translocation and induction of cytokine genes. CN activity can be inhibited using the commercially available immunosuppressants, tacrolimus and cyclosporine. Physical interactions between CN and NFATs can be blocked using peptide based reagents like VIVIT. Many cytokines that are induced by the CN/NFAT pathway can stimulate astrocytes in an autocrine or paracrine manner, triggering elevations in intracellular Ca^2+^, which can lead to further CN activation. Severe Ca^2+^ dysregulation can convert CN into a constitutively active proteolytic fragment (ΔCN) via calpain dependent proteolysis. Hyperactivation of CN/NFAT maintains chronic neuroinflammation (and astrocyte activation) through continued induction (i.e., a positive feedback loop) of pro-inflammatory cytokine genes.

The CN holoenzyme consists of a catalytic subunit and a Ca^2+^-binding regulatory subunit (Norris, 2014). The catalytic subunit contains a critical autoinhibitory domain (AID) and a calmodulin binding site. Ca^2+^/calmodulin binding to CN is the primary stimulus for driving maximal CN phosphatase activity (Figure 1). When cellular Ca^2+^ levels are low, the AID masks the catalytic core and maintains CN in an inactive state. Cooperative binding of Ca^2+^ to the CN regulatory subunit and to calmodulin lead to the rapid displacement of the AID and robust activation of CN. When Ca^2+^ levels fall, Ca^2+^/calmodulin rapidly dissociates from CN, reinstating inhibition by the AID. CN is highly sensitive to Ca^2+^, with a Kd to Ca^2+^-saturated calmodulin in the picomolar range (Quintana et al., 2005). Thus, even small perturbations in cellular Ca^2+^ can lead to hyperactivation of CN. Under these conditions, CN activity can still be normalized if Ca^2+^ levels recover. However, large surges in Ca^2+^ can trigger the calpain-mediated proteolytic removal or disruption of the CN AID. Without the AID, CN becomes partially (but permanently) uncoupled from local Ca^2+^ changes and exhibits constitutively high levels of activity (i.e., in the presence or absence of Ca^2+^). Appearance of the CN proteolytic fragment (ΔCN) is one of the most clear-cut indicators of hyperactive CN signaling (Figure 1). Many commercial CN antibodies (directed to the CN carboxyl terminus) do not detect ΔCN in Western blot applications, which may explain why earlier studies failed to observe elevated CN in neurodegenerative conditions like AD (Gong et al., 1993; Ladner et al., 1996; Lian et al., 2001). In contrast, more recent work (using N terminus antibodies) has found that ΔCN is increased in human AD tissue (Liu et al., 2005; Wu et al., 2010; Mohmmad Abdul et al., 2011; Watanabe et al., 2015), in parallel with calpain activation (Liu et al., 2005; Mohmmad Abdul et al., 2011). The ΔCN fragment has been reported in numerous other experimental models of brain injury and disease including traumatic brain injury (TBI), ischemia and glaucoma (Norris, 2014).

## CN Expression Is Increased in Activated Astrocytes in Humans and Animal Models

Cell-specific expression patterns of CN in both humans and animal models can exhibit striking changes characterized by intense upregulation in subsets of activated astrocytes (Hashimoto et al., 1998; Celsi et al., 2007; Abdul et al., 2009; Lim et al., 2013; Liu et al., 2015; Watanabe et al., 2015; Pleiss et al., 2016b; Sompol et al., 2017). Recent work using custom antibodies generated toward calpain-dependent proteolysis sites in the CN catalytic subunit, observed extensive labeling for a 45–48 kDa ΔCN proteolytic fragment in astrocytes and, to a seemingly lesser extent, neurons (Pleiss et al., 2016b). ΔCN was especially prominent in activated astrocytes bordering amyloid deposits and microinfarcts in human specimens (Pleiss et al., 2016b). Interestingly, ΔCN-positive and ΔCN-negative astrocytes were often found in the same regions (sometimes side-by-side) and appeared morphologically similar, highlighting the biochemical heterogeneity of activated astrocytes. In an aggressive mouse model of AD (i.e., 5xFAD mice), ΔCN was similarly observed in activated astrocytes in the hippocampus, increasing in direct proportion to elevated GFAP levels (Sompol et al., 2017). These observations are consistent with previous reports that found high levels of calpain activity in activated astrocytes (Shields et al., 1998, 2000; Feng et al., 2011) and suggest that Ca^2+^ dependent proteolysis of CN is a major outcome of astrocytic Ca^2+^ dysregulation.

## NFATs

There are five primary NFAT family members: NFAT1 (or NFATp, NFATc2), NFAT2 (or NFATc, NFATc1), NFAT3 (or NFATc4), NFAT4 (or NFATc3) and NFAT5, all of which exhibit DNA-binding domains that are structurally similar to the Rel/NFκB family of transcription factors (Rao et al., 1997). Elevations in Ca^2+^ activate CN, which binds to and dephosphorylates NFATs 1–4 in the cytosol (Figure 1). NFAT5 is activated by osmotic stress and does not interact with CN. Dephosphorylation of NFATs exposes a nuclear localization signal, leading to transport into the nucleus and interaction with specific DNA binding elements. Similar to CN, NFAT activation is typically elevated under neurodegenerative conditions like AD (Abdul et al., 2009; Wu et al., 2010), Parkinson’s disease (Caraveo et al., 2014), and acute brain injury (Serrano-Pérez et al., 2011; Furman et al., 2016). As with other previously mentioned transcription factors (e.g., NFkB, JAK-STAT, AP-1,…etc), NFATs exert broad control over several transcriptional programs via the up- and downregulation of numerous genes, many of which involve cytokines and other classic inflammatory mediators (Im and Rao, 2004; Figure 1). NFATs are very strongly inhibited by the CN-inhibiting drugs tacrolimus and cyclosporine, but can be specifically targeted by a variety of peptide-based reagents. The VIVIT peptide, based on the endogenous CN docking sequence (PxIxIT) located in the N terminus of the regulatory region of NFATs 1–4, prevents CN from binding to and dephosphorylating NFATs (Aramburu et al., 1999). Thus, unlike commercial CN inhibitors, VIVIT impairs CN-mediated activation of NFATs without inhibiting CN activity *per se*, providing a powerful reagent for teasing apart NFAT-dependent signaling from the broader NFAT-independent actions of CN (Figure 1).

In peripheral tissues, NFATs play key roles in phenotype switching. Activation/anergy of T lymphocytes (Hogan, 2017), myotube formation and fiber-type commitment (Horsley and Pavlath, 2002; McCullagh et al., 2004; Rana et al., 2008), cardiomyocyte hypertrophy (Molkentin, 2004), vascular smooth muscle cell migration and proliferation (Liu et al., 2004; Karpurapu et al., 2010; Kundumani-Sridharan et al., 2013), and bone and joint remodeling (Sitara and Aliprantis, 2010) all depend critically on the NFAT pathway. Though not as extensively investigated in the CNS, several studies suggest that NFATs play a key role in the activation of astrocytes and microglia, as well (Nagamoto-Combs and Combs, 2010; Furman and Norris, 2014). All four CN-dependent NFATs have been identified in primary astrocytes at the mRNA and protein levels (Canellada et al., 2008). NFAT1 was found at higher levels in astrocyte nuclei in postmortem brain sections taken from human subjects with mild cognitive impairment (Abdul et al., 2009). NFAT1 has also been identified in microglia of AD mouse models (Manocha et al., 2017b). However, relative to all other NFAT isoforms, NFAT4 appears to show the greatest association with astrocytes in intact animals, with comparatively much less expression in neurons (Filosa et al., 2007; Serrano-Pérez et al., 2011; Neria et al., 2013; Caraveo et al., 2014; Yan et al., 2014; Furman et al., 2016; Sompol et al., 2017). While one study observed a reduction in NFAT4 protein levels in rats exposed to TBI (Yan et al., 2014), several other studies found that NFAT4 is strongly induced in activated astrocytes as a result of acute injury or progressive amyloid or synuclein pathology (Serrano-Pérez et al., 2011; Neria et al., 2013; Caraveo et al., 2014; Furman et al., 2016; Sompol et al., 2017).

## Glial CN/NFAT Pathway and Neuroinflammatory Signaling

Similar to actions in lymphocytes, glial CN/NFAT activity appears to play a critical role in regulating immune/inflammatory responses. In primary astrocytes and microglia, the CN/NFAT pathway is robustly activated by many key inflammatory mediators, including cytokines, Aβ, glutamate and vascular injury-associated factors (Fernandez et al., 2007; Canellada et al., 2008; Pérez-Ortiz et al., 2008; Sama et al., 2008; Abdul et al., 2009; Furman et al., 2010; Nagamoto-Combs and Combs, 2010; Rojanathammanee et al., 2015). Little is known about the Ca^2+^ sources that are responsible for glial CN activation but L-type voltage sensitive Ca^2+^ channels have been specifically implicated in astrocytes (Canellada et al., 2008; Sama et al., 2008). Overexpression of the hyperactive ΔCN fragment in astrocytes leads to the upregulation of numerous immune/inflammatory related genes (Norris et al., 2005; Fernandez et al., 2007) and functional gene categories linked to the activated astrocyte phenotype (i.e., morphogenesis, cell adhesion and immune response; Norris et al., 2005). Interestingly, many of the genes identified in Norris et al. (2005) are part of the A1 “neurotoxic” astrocyte transcriptional signature described by the Barres lab (Zamanian et al., 2012; Liddelow et al., 2017). Of note, ΔCN triggered a two-to-three fold increase in the A1-associated complement component C3, found recently to drive microglia-mediated synapse loss in mouse models of AD (Hong et al., 2016; Shi et al., 2017). In addition to CN-activation studies, inhibitory approaches in primary cultures have revealed similar roles for CN/NFATs in neuroinflammation. Immune/inflammatory factors sensitive to CN/NFAT inhibition in glial cells include TNFα, GM-CSF, IL-6, CCL2 and Cox2, among others (Canellada et al., 2008; Sama et al., 2008; Nagamoto-Combs and Combs, 2010; Kim et al., 2011; Watanabe et al., 2015; Manocha et al., 2017b).

Bidirectional interactions between CN/NFAT and cytokine factors suggest that the CN/NFAT pathway is ideally suited to maintain positive feedback cycles underlying chronic neuroinflammation (Griffin et al., 1998; Figure 1). Consistent with this possibility, hyperactive CN/NFAT activity has been shown to propagate across local astrocyte networks through a paracrine signaling mechanism (Sama et al., 2008). A significant question remains about the mechanisms that keep these feedback cycles in check. One possibility is that CN/NFAT activity is limited by the expression of endogenous CN inhibitors. Regulator of CN 1 (RCAN1), for instance, is strongly induced by NFAT activity in multiple cell types including astrocytes (Canellada et al., 2008; Sobrado et al., 2012). RCANs are widely considered as CN inhibitors, though, it deserves noting that several studies have revealed permissive effects of RCAN on CN, depending on the presence of key accessory proteins (Liu et al., 2009). Whether RCANs provide a negative feedback mechanism for guarding against progressive Ca^2+^ dysregulation and neuroinflammation in astrocytes, in the context of neurodegeneration, will require further investigation.

Finally, caution should be taken when interpreting immune/inflammatory actions of CN/NFATs in primary glia which are very sensitive to culturing conditions. When investigated in serum-containing media, primary astrocytes may exhibit a quasi-activated state characterized by elevated basal levels of CN/NFAT activity (Furman et al., 2010). Indeed, addition of standard (10% fetal calf) serum alone induces robust CN/NFAT activity in primary astrocytes previously maintained in serum-free media (Furman et al., 2010). Moreover, treatment with IL1-β, IF-γ, or TNFα, which strongly induce NFAT activity in the absence of sera, elicited significantly muted responses when delivered in the presence of sera. Similar caution is also warranted in studies on intact animals, where the effects of CN/NFAT inhibition may have very different effects on glial activity and neuroinflammation, depending on the nature of the insult. For instance, intracerebroventricular delivery of the VIVIT peptide, or astrocyte-specific expression of VIVIT using adeno-associated virus (AAV), reduced signs of astrocyte and microglial activation in mouse models of AD characterized by progressive amyloid pathology (Abdul et al., 2010; Furman et al., 2012; Rojanathammanee et al., 2015; Sompol et al., 2017), but not in a rat model of TBI characterized by acute trauma (Furman et al., 2016). The reason for these discrepancies is unclear, but could involve CN/NFAT interactions with multiple other transcription factors and signaling pathways (as discussed further below). In any case, the results highlight the importance of context in understanding astrocytic CN/NFAT signaling.

## Astrocytic CN/NFAT Pathway in Glutamate Dysregulation

Mounting evidence suggest that activated astrocytes may lose protective glutamate buffering properties in some forms of injury and disease. Astrocytes control extracellular glutamate levels, in part, through the use of several excitatory amino acid transporters (EAATs) located in the astrocyte plasmalemma. The EAAT2/GLT-1 protein is responsible for the bulk of glutamate uptake in several brain regions, including hippocampus (Robinson and Jackson, 2016). Loss of EAAT2 has been observed in several human neurodegenerative conditions including AD (Masliah et al., 1996; Abdul et al., 2009; Simpson et al., 2010), Alexander disease (Tian et al., 2010), epilepsy with hippocampal sclerosis (Mathern et al., 1999; Proper et al., 2002), and TBI (van Landeghem et al., 2006). Similar changes have been reported in corresponding animal models (Masliah et al., 2000; Mookherjee et al., 2011; Schallier et al., 2011; Hefendehl et al., 2016; Sompol et al., 2017). Functional knockdown of EAAT2/GLT-1 very typically causes synaptic hyperexcitability, altered synaptic plasticity, excitotoxicity and a variety of functional deficits depending on the brain region affected (Rothstein et al., 1996; Rao et al., 2001; Selkirk et al., 2005; Petr et al., 2015; Moidunny et al., 2016). In contrast, increased expression/function of EAAT2/GLT-1 provides strong neuroprotection from exogenously delivered excitotoxins as well as from acute and chronic CNS injury and disease (Harvey et al., 2011; Rozkalne et al., 2011; Zumkehr et al., 2015; Karklin Fontana et al., 2016).

The human EAAT2 promoter has putative binding sites for numerous transcription factors linked to neuroinflammation, including NFATs (Kim et al., 2003; Su et al., 2003; Mallolas et al., 2006), and is activated (and in some cases, inhibited) by a number of cytokine factors. Several studies suggest that the CN/NFAT pathway provides a putative link between Ca^2+^ dysregulation, neuroinflammation and glutamate dysregulation in activated astrocytes through modulation of EAAT/GLT-1 expression. Recent work found that overexpression of the ΔCN fragment significantly reduced EAAT-mediated glutamate uptake in primary astrocytes (Sompol et al., 2017). In contrast, inhibition of CN/NFAT activity with the VIVIT peptide protected EAAT2-GLT-1 protein levels and reduced extracellular glutamate and/or neuronal hyperexcitability in primary cultures following treatment with either IL1-β or oligomeric Aβ (Sama et al., 2008; Abdul et al., 2009). Under the same treatment conditions, significantly greater neuronal survival was observed when astrocytic CN/NFAT activity was inhibited with VIVIT. Similar effects were found following VIVIT treatment in an intact mouse model of AD (Sompol et al., 2017). Specifically, VIVIT increased protein levels of the astrocytic glutamate transporter, GLT-1, especially around Aβ deposits, and reduced the frequency and duration of spontaneous glutamate transients in intact 5xFAD mice. VIVIT also quelled hyperactive synaptic transients in *in situ* brain slices from 5xFAD mice and reduced the augmented NMDA receptor-mediated component of basal synaptic transmission. The reduction in glutamate hyperexcitability in 5xFAD mice was accompanied by the normalization of dendrite morphology and integrity, suggesting that astrocyte activation and astrocytic CN/NFAT signaling can drive excitotoxic damage in some disease states, like AD.

## Astrocytic CN/NFAT Pathway in Amyloid Pathology

Amyloid pathology has long been recognized as a potent stimulus for CN and/or NFAT activity in multiple neural cell types (Agostinho et al., 2008; Reese et al., 2008; Abdul et al., 2009; Li et al., 2010; Wu et al., 2010, 2012; Mohmmad Abdul et al., 2011; Fang et al., 2016). Mice with parenchymal amyloid pathology show clear Ca^2+^ dysregulation in astrocytes: i.e., higher basal Ca^2+^ levels and bigger and more frequent Ca^2+^ transients (Kuchibhotla et al., 2009), providing a permissive environment for CN/NFAT activity. In human postmortem tissue, elevations in CN/NFAT activity increase in direct proportion with soluble Aβ levels, within the same subjects (Abdul et al., 2009). In primary neuron/astrocyte cultures, Aβ stimulates CN/NFAT activity and generates ΔCN proteolytic fragments (Mohmmad Abdul et al., 2011). Moreover, CN/ΔCN is found at especially high levels in activated astrocytes surrounding amyloid deposits in both mouse and human tissue (Norris et al., 2005; Celsi et al., 2007; Abdul et al., 2009; Jin et al., 2012; Lim et al., 2013; Watanabe et al., 2015; Pleiss et al., 2016b).

In addition to responding to Aβ, several studies have suggested that astrocytic CN/NFAT activity stimulates the generation of Aβ peptides (Hong et al., 2010; Furman et al., 2012; Jin et al., 2012; Sompol et al., 2017). Peripheral administration of the CN inhibitor, tacrolimus, to 8-month-old APP/PS1 transgenic mice over a period of 2 months led to a large (>75%) significant reduction in amyloid plaque burden in both the hippocampus and cortex (Hong et al., 2010). A smaller (20%–30%), but statistically significant decrease in amyloid plaque load and soluble Aβ peptide levels was also observed when CN/NFAT activity was specifically inhibited in hippocampal astrocytes of 2x and 5xAPP/PS1 mice using AAV-mediated delivery of VIVIT (Furman et al., 2012; Sompol et al., 2017). Though reductions in Aβ could have simply stemmed from the increased viability of neurons in tacrolimus/VIVIT treated mice, an additional report by Sompol et al. (2017) demonstrated that Ca^2+^ overload can lead to elevated Aβ production—specifically within astrocytes—through a CN/NFAT4-dependent mechanism. In this study, NFAT4 was shown to bind to the promoter of BACE1 (the rate limiting enzyme for Aβ generation) and induce BACE1 transcription. These results suggest that astrocytic CN/NFATs may help to drive parenchymal Aβ plaque pathology in AD. Given the intimate association between astrocytes and the cerebrovasculature, it would be interesting to determine if astrocytic CN/NFATs play a particularly important role in cerebral amyloid angiopathy.

## Astrocytic CN/NFAT Pathway in Synapse Dysfunction

As discussed, commercial CN inhibitors are commonly associated with neuroprotective, anti-inflammatory and nootropic properties across a wide-range of experimental models of neural injury and disease. Within our lab, synaptoprotection has emerged as the single most consistent functional outcome of inhibiting CN/NFAT activity in astrocytes. To inhibit CN/NFATs, we have relied heavily on AAV vectors expressing the NFAT inhibitor, VIVIT, under the control of the human GFAP promoter (Gfa2). Delivery of AAVGfa2-VIVIT to the hippocampus of adult rodents results in widespread, astrocyte-selective transgene expression, coincident with the inhibition of NFAT4 nuclear translocation (Furman et al., 2012, 2016; Sompol et al., 2017). AAV-Gfa2-VIVIT improves basal hippocampal synaptic strength in double transgenic APP/PS1 transgenic mice (Furman et al., 2012), 5xFAD mice (Sompol et al., 2017), rats with TBI (Furman et al., 2016), and mice with hyperhomocysteinemia (HHcy)-associated vascular pathology (Pleiss et al., 2016a). In regards to synaptic plasticity, AAV-Gfa2-VIVIT improves long-term potentiation (LTP) in double transgenic APP/PS1 mice (Furman et al., 2012) and suppresses the induction of long-term depression in TBI rats (Furman et al., 2016). Investigations on LTP in HHcy mice have shown very similar outcomes. In contrast, hyperactivation of CN in astrocytes of otherwise healthy adult rats, using AAV-Gfa delivery of the ΔCN fragment, induces local deficits in CA3-CA1 synaptic strength (Pleiss et al., 2016b). Though not investigated in every study, we have also found that delivery of AAV-Gfa2-VIVIT to hippocampal astrocytes of AD mouse models improves hippocampal-dependent cognition (Furman et al., 2012; Sompol et al., 2017).

**Figure 2:**
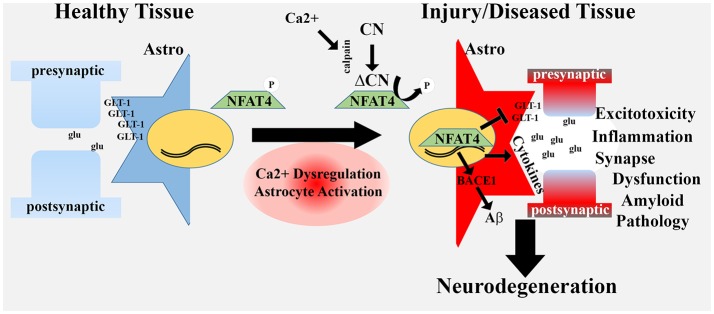
Hyperactivated CN/NFAT signaling in astrocytes may give rise to a neurotoxic astrocyte phenotype. In healthy tissue, astrocytes fine-tune synaptic communication and protect neuronal viability through numerous mechanisms, including uptake of excitotoxic glutamate (glu) at synapses, via GLT-1 transporters. During aging, injury and disease, many astrocytes exhibit an activated phenotype that includes Ca^2+^ dysregulation, proteolysis of CN to a high activity fragment (ΔCN) and induction of the NFAT4 isoform. Hyperactivation of NFAT4 leads to the downregulation of GLT-1, production and release of numerous pro-inflammatory cytokines, and induction of BACE1. These changes underlie a neurotoxic astrocyte phenotype associated with glutamate dysregulation/excitotoxicity, neuroinflammation, synapse dysfunction and amyloid pathology. Neurotoxic astrocytes contribute to or hasten neurodegenerative processes leading to dementia.

It is presently unclear how or why astrocytic CN/NFAT signaling negatively affects synapses. Many of the CN-dependent cytokines released from astrocytes are known to disrupt synaptic viability under certain conditions. In fact, several cytokine-inhibiting drugs appear to have remarkably similar effects to astrocyte-VIVIT treatment in AD mouse models (Kotilinek et al., 2008; Bachstetter et al., 2012; MacPherson et al., 2017). In addition, CN-dependent TGF-β release from astrocytes was recently found to suppress PSD-95 levels in nearby neurons (Tapella et al., 2018). In addition to cytokines, gene microarray studies in primary cells and protein measurements in TBI rats suggest that CN/NFATs drive the induction of factors involved in synapse turnover and/or remodeling, including complement cascade components (e.g., C3) and matricellular factors (e.g., SPARC and hevin; Norris et al., 2005; Furman et al., 2016). As mentioned, C3 was recently identified as a key component of the “neurotoxic” A1 activated astrocyte phenotype (Liddelow et al., 2017). During development, C3 release from astrocytes tags synapses for microglia-mediated phagocytosis, leading to synapse removal/remodeling (Stevens et al., 2007). C3 levels drop during maturation, but then reappear under pathological conditions, like AD (Eikelenboom and Veerhuis, 1996; Zabel and Kirsch, 2013). Recent work found that C3 upregulation in activated astrocytes in an APP/PS1 mouse model of AD guides microglia-mediated synapse loss, similar to that observed during development (Lian et al., 2015; Hong et al., 2016; Shi et al., 2017). In the Lian et al.’s (2015) study, C3 induction in astrocytes was attributable to the activation of NFκB (which can be activated by CN, see below), though a role for NFAT was not investigated. The matricellular proteins SPARC and hevin are also developmentally regulated factors that become induced in activated astrocytes in mature brain following injury and/or disease (Jones and Bouvier, 2014; Blakely et al., 2015; Furman et al., 2016). These factors regulate adhesion and de-adhesion of astrocytes with the extracellular matrix where they influence interactions with the vasculature, with other astrocytes, and also with neurons, especially at synapses, leading to synaptogenesis and re-modeling (Jones et al., 2011; Kucukdereli et al., 2011; Jones and Bouvier, 2014; Blakely et al., 2015). Hevin, a pro-synaptogenic factor, is very strongly induced in TBI rats treated with AAV-Gfa2-VIVIT, suggesting that activated astrocytes and hyperactive CN/NFAT signaling inhibit the formation of new synapses by suppressing hevin levels, at least in the context of acute neural injury (Furman et al., 2016). Finally, it seems likely that glutamate dysregulation and Aβ pathology play a significant and non-specific role in synapse dysfunction. Indeed, synapses are very sensitive to excitotoxic insults and circulating oligomeric Aβ peptide levels. By contributing to glutamate dysregulation and amyloid toxicity, activated astrocytes and hyperactive CN/NFAT signaling may simply promote an inhospitable working environment for synapses. Of course, all of these mechanisms could be working in concert as part of a broader neurotoxic astrocyte phenotype, with Ca^2+^ dysregulation and hyperactive CN/NFAT4 activity as central driving features (Figure 2).

## Non-NFAT Targets of CN in Astrocytes and Current Controversies

NFATs may be the most studied, but they are certainly not the only substrates for CN. In fact, CN has been shown to interact with most transcription factors involved in immune/inflammatory signaling. NFκB, for instance, is strongly regulated by CN activity, though in a fairly indirect manner. CN does not appear to physically bind to or dephosphorylate NFκB, but instead interacts with upstream targets that drive NFκB activation (Pons and Torres-Aleman, 2000; Frischbutter et al., 2011; Palkowitsch et al., 2011). CN-dependent activation of NFκB in astrocytes has been shown to modulate the expression of immune/inflammatory genes (Fernandez et al., 2007) and genes involved in Ca^2+^ signaling and homeostasis (e.g., mGluR5 type glutamate receptors and inositol triphosphate (IP3)-dependent Ca^2+^ release channels; Lim et al., 2013). IP3-receptors play an important role in regulating intracellular Ca^2+^ transients and waves in astrocytes (Filosa et al., 2004; Wu et al., 2017) and have been suggested to mediate neurotoxic actions of activated astrocytes in Alexander disease (Saito et al., 2018). CN/NFκB-dependent upregulation of mGluR5 and IP3 receptors occurs in direct response to pathogenic Aβ peptides and provides an intriguing CN-based mechanism for driving astrocytic Ca^2+^ dysregulation in AD mouse models (Kuchibhotla et al., 2009).

In addition to NFATs and NFκB, recent work suggests that CN can exert transcriptional control in astrocytes through novel interactions with the forkhead transcription factor, FOX03 (Fernandez et al., 2012, 2016). Proinflammatory cytokines, like TNFα, or Aβ peptides, stimulated the physical association between CN and FOX03, leading to dephosphorylation of FOXO3 and association with NFκB. The CN/FOX03/NFκB complex is thought to drive gene programs underlying deleterious neuro-immune/inflammatory signaling. Using an approach similar to the VIVIT strategy for inhibiting CN-NFAT interactions, Fernandez et al. (2016) developed a mimetic peptide that selectively disrupts CN-FOXO3 interactions. When delivered to primary astrocytes the CN-FOX03 interfering peptide reduced Aβ production and reduced the expression of pro-inflammatory cytokines. Interestingly, treatment of astrocytes with the neurotrophic factor, insulin like growth factor 1 (IGF-1), inhibited CN/FOX03/NFκB interactions and instead promoted the association of CN with NFκB and the peroxisome proliferator-activated receptor-γ (PPAR-γ). Formation of the CN/PPAR-γ/NFκB complex in astrocytes was associated with reduced amyloid pathology and improved cognitive function in an AD mouse model (Fernandez et al., 2012). These results suggest that CN activation in astrocytes can drive either deleterious or protective processes depending on which transcription factors are engaged. This work is consistent with other studies that find both beneficial and detrimental actions of activated astrocytes in disease models (Pekny and Pekna, 2014; Pekny et al., 2016). Moreover, there is good precedence for divergent actions of CN on gene expression programs in other non-neural cell types. For instance, CN activation in T lymphocytes can drive or inhibit expression of immune/inflammatory factors by interacting with different transcription factors in different T cell subtypes or in response to changing environmental conditions (Im and Rao, 2004; Wu et al., 2006).

Interestingly, overexpression of a ΔCN proteolytic fragment in astrocytes using a GFAP promoter was shown to have similar effects as IGF-1 stimulation, yielding beneficial effects in an AD mouse model, and in mice exposed to acute stab wound or LPS insult (Fernandez et al., 2007, 2012). The mechanisms of ΔCN’s beneficial effects are unclear. It is unknown whether ΔCN interacts with the PPARγ/NFκB complex, or if ΔCN opposes the interaction of NFκB with FOXO3, or if NFATs are involved in any of these pathways. The beneficial effects of ΔCN from the Fernandez et al. (2007, 2012) studies are especially unusual as this fragment is largely uncoupled from its normal mode of regulation (Ca^2+^/calmodulin) and is most commonly associated with cellular dysfunction and cell death in many different cell types, though there are some rare exceptions e.g., (Bousette et al., 2010). These results are also in apparent contrast to recent work showing that ΔCN expression in healthy rats drives (rather than prevents) local synapse dysfunction (Pleiss et al., 2016b). An alternative possibility for ΔCN-mediated neuroprotection in the Fernandez et al. (2007, 2012) studies may relate to an interaction between existing brain pathology and the over-expression system used (i.e., genetically modified ΔCN under the control of a GFAP promoter). Preexisting injury or amyloid pathology may be expected to strongly induce the GFAP promoter, leading to the intense upregulation of ΔCN in target cells, which could, possibly, lead to the death/deterioration of the most reactive and/or the most harmful astrocytes. Loss of harmful astrocytes may ultimately improve the viability of nearby neurons. Clearly, further research will be necessary to test this possibility.

Finally, CN is a versatile enzyme with numerous functions that are independent of transcriptional regulation. Nonetheless, very few non-transcription factor substrates of CN have been investigated in astrocytes. In most cases, CN’s interactions with other targets has been implied based on sensitivity to commercial CN inhibitors. For instance, tacrolimus and cyclosporine partially blocked the dephosphorylation of GFAP and vimentin in primary astrocytes and in brain slices from neonatal rat pups (Vinadé et al., 1997; Carvalho et al., 2016), suggesting that CN may regulate astrocyte morphology through a posttranscriptional mechanism. These results are reminiscent of studies in neurons, where CN has been long-known to regulate rapid cytoskeletal reorganization in dendritic spines and growth cones (Halpain et al., 1998; Wen et al., 2004). Given the dynamic nature of astrocyte processes and endfeet, it seems likely that CN would play a similar role in cytoskeletal reorganization in astrocytes. In addition to intermediate filaments, the astrocyte hemichannel protein, connexin 43, has also been revealed as a potential CN substrate (Li and Nagy, 2000; Tence et al., 2012). The cytoplasmic tail of connexin 43 is dephosphorylated in a tacrolimus/cyclosporine sensitive manner during hypoxic/ischemic insults. Interestingly, this dephosphorylation was associated with reduced gap junction coupling, which could have important implications for potassium and glutamate buffering during neural injury and disease. And, as with many other cell types, mitochondria function in astrocytes appears to be very sensitive to tacrolimus/cyclosporine (Kahraman et al., 2011; O’Donnell et al., 2016), though, it should be noted that cyclosporine can inhibit formation of the mitochondrial transition pore in a CN-independent manner (Halestrap et al., 1997). In one recent study, both tacrolimus and cyclosporine prevented the loss of mitochondria from astrocyte processes during hypoxic/ischemic insult (O’Donnell et al., 2016). However, it remains unclear how CN specifically contributed to this loss.

## Summary

Mounting evidence suggests that the CN/NFAT pathway links astrocytic Ca^2+^ dysregulation to molecular and phenotypic changes involved with neuroinflammation, glutamate dysregulation, amyloid pathology and synapse dysfunction. We hypothesize that the increased expression and/or hyperactivation of CN/NFAT in activated astrocytes—found in human neurodegenerative disease and animal models of disease—plays a predominantly deleterious role in the brain, arising early in neurodegenerative diseases, like AD, and progressing as disease symptoms worsen (Figure 2). The numerous beneficial effects reported in disease models treated with CN and/or NFAT inhibitors is largely consistent with this hypothesis. These observations provide a very important extension and/or reconceptualization of the Ca^2+^ hypothesis of aging and disease to include glial Ca^2+^ dyshomeostasis and altered CN signaling as a critical component in the initiation and progression of neurodegeneration. Further work will be needed to tease apart the actions of CN on different transcriptional pathways and how these pathways interact to modulate neural function in healthy and diseased brain.

## Author Contributions

PS and CN researched and wrote this manuscript.

## Conflict of Interest Statement

The authors declare that the research was conducted in the absence of any commercial or financial relationships that could be construed as a potential conflict of interest.
